# Hemolymph composition, gene expressions in the gills, and thus the survival of euryhaline crabs are controlled by ambient minor cations according to osmotic condition‐dependent manner

**DOI:** 10.1002/ece3.6846

**Published:** 2020-10-07

**Authors:** Masahiro Yamaguchi, Kouichi Soga

**Affiliations:** ^1^ Department of Chemistry and Biochemistry National Institute of Technology Suzuka College Suzuka Japan; ^2^ Department of Biology Graduate School of Science Osaka City University Osaka Japan

**Keywords:** ambient minor cations, euryhaline crabs, gills, hemolymph, osmolality

## Abstract

Na^+^ and Cl^−^ are the most abundant dissolved ions in seawater, constituting ~ 85% of total ions. They significantly affect the osmolality of body fluids of marine invertebrates. Seawater also contains minor ions such as Mg^2+^, Ca^2+^, K^+^, and SO_4_^2‐^ , but their effects on marine organisms are unclear. This study analyzed the effects of Mg^2+^, Ca^2+^, and K^+^ (ambient minor cations) on survival, hemolymph ionic composition, and gene expression in the gills of three euryhaline crabs: *Helice tridens*, *Macrophthalmus japonicus*, and *Chiromantes dehaani*. Ambient minor cations were required for survival of *H. tridens* and *M. japonicus* under isosmotic conditions with seawater. The ambient minor cations also affected the osmolality and ionic composition of hemolymph by regulating expressions of specific genes in the gills required for Na^+^ uptake, such as Na^+^/K^+^ ATPase, cytoplasmic carbonic anhydrase, and Na^+^/H^+^ exchanger. Administration of carbonic anhydrase and Na^+^/H^+^ exchanger inhibitors increased the survival rate even if ambient minor cations did not exist. In contrast, under hypo‐osmotic conditions, ambient minor cations had different effects on crabs, a lethal effect on *M. japonicus*, and an increase of the hemolymph K^+^ concentration in *H. tridens* and *M. japonicus*. It is thus concluded that the effects of ambient minor cations are osmolality‐dependent. In contrast, in *C. dehaani*, the hemolymph ionic composition and survival rate were hardly affected by ambient minor cations, probably reflecting the habitat of this species. These results strongly indicated that *C. dehaani* is less susceptive to ambient minor cations compared to *H. tridens* and *M. japonicus*.

## Graphical abstract

In this study, effects of ambient minor cations on three species of euryhaline crabs, *Helice tridens*, *Macrophthalmus japonicus*, and *Chiromantes dehaani*, were studied. We revealed that survival, hemolymph ionic composition, and gene expressions involved in Na^+^ uptake were affected by minor cations in *H. tridens* and *M. japonicus*. On the contrary, *C. dehaani*, which is most adaptive to fresh water, was less susceptible to minor cations.

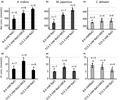

## 1 INTRODUCTION

Dissolved ions in seawater play a crucial role in maintaining the osmolality of body fluids of marine invertebrates. Na^+^ and Cl^−^ are the most abundant ions in seawater, constituting ~85% of total ions, in addition to minor ions such as SO_4_^2‐^, Mg^2+^, Ca^2+^, and K^+^. If dissolved ions are required only for maintaining the osmolality of body fluids, minor ions should not be essential and their replacement by Na^+^ and Cl^−^ should have no effect on the life of marine invertebrates. However, the euryhaline prawn *Penaeus mondon* cannot survive in 0.17% NaCl solution, although it can survive in diluted artificial seawater of the same salinity (Cawthorne et al., 1983). In addition, the presence of K^+^, Mg^2+^, and SO_4_^2‐^ increases the survival rate of euryhaline prawns *Litopenaeus vannamei* and *Melicertus latisulcatus* (formerly called *P. latisulcatus)* (Davis et al., 2005; Prangnell & Fotedar, 2005, 2006; Roy et al., 2007; Saoud et al., 2003; Zhu et al., 2004). Therefore, minor ions are required for the survival of marine invertebrates, at least for some euryhaline crustaceans. However, the mechanisms by which minor ions affect the survival of marine organisms are unclear.

Most crustacean species inhabiting estuaries are osmoregulators; they can regulate hemolymph osmotic and ionic concentrations in response to ambient salinity changes to some extent. Typical osmoregulation in estuarine crustaceans is hyper‐isosmotic, with hyperregulation in low salinity and isosmotic regulation in salinity close to or higher than seawater (Charmantier et al., 2009; Lignot & Charmantier, 2015). Such osmoregulatory pattern allows crustacean species in estuaries to be euryhaline and tolerate a wider range of ambient salinity. Many studies have investigated the mechanisms by which euryhaline crabs exposed to fresh/brackish water maintain their hemolymph osmolality higher compared to the environment, and chloride cells in posterior gills play an important role in hemolymph osmotic and ionic regulation (Freire et al., 2008; Henry et al., 2012). Chloride cells express Na^+^/K^+^ ATPase (NKA) in the basolateral membrane, which transports Na^+^ from the cytoplasm to the hemolymph and maintains intracellular Na^+^ concentration relatively low. Another key enzyme in chloride cells is cytoplasmic carbonic anhydrase (CAc), which catalyzes the formation of H^+^ and HCO_3_^‐^ from H_2_O and CO_2_, and the derived H^+^ supports Na^+^ uptake through activation of the Na^+^/H^+^ exchanger (NHE) located in the apical membrane. Other molecules, such as V‐type H^+^ ATPase and Na^+^/K^+^/2Cl^−^ cotransporter (NKCC), are also involved in regulating salinity and ionic composition of hemolymph (Charmantier et al., 2009; Freire et al., 2008; Griffith, 2017; Henry et al., 2012). Transcriptome analysis has identified numerous genes whose expression levels are modified by changes in ambient salinity (Lv et al., 2013; Towle et al., 2011), and these genes might also contribute to hemolymph osmotic and ionic regulation. However, it is unknown at present what factor(s) in environmental water are used as key signal of ambient salinity, and what factor(s) induce changes of the gene expressions in the gills.

This study analyzed the effects of Mg^2+^, Ca^2+^, and K^+^ (hereafter called “ambient minor cations”) on survival, hemolymph ionic composition, and gene expression in the gills of three euryhaline crabs: *Helice tridens*, *Macrophthalmus japonicus*, and *Chiromantes dehaani*. The different response to ambient minor cations between euryhaline species could be due to the difference in the microenvironment of their habitats or phylogeny.

## 2 MATERIALS AND METHODS

### 2.1 Animals and evaluation of survival rates in various bathing media

*Helice tridens* and *M. japonicus* were collected from the Tanaka river estuary in Tsu City. *Chiromantes dehaani* was collected from the Suzuka river estuary in Yokkaichi City, Mie Prefecture, Japan. In order to examine the effects of ionic composition of bathing media on survival rate, four individuals of each species were reared in 210 × 300 × 95 mm plastic tanks with 1,000 ml of bathing media (for *H. tridens* and *C. dehaani*), or in 177 × 195 × 100 mm plastic tanks with 500 ml of bathing media (for *M. japonicus*). The compositions of bathing media used in this study were summarized in Table 1. The tanks were kept in an incubator in a 16 hr/8 hr light/dark cycle at 21°C. The survival rate of crabs was assessed at 16, 24, 40, and 48 hr, and carcasses were removed at each time point. The survival rate at 48 hr was calculated and presented unless otherwise noted. The experiments were performed at least in triplicate.

**Table 1:** The composition of bathing media used in this study

Solution name	NaCl	MgSO_4_	MgCl_2_	CaCl_2_	KCl	Osmotic concentration
513.3 mmol/L NaCl	513.3					949
256.7 mmol/L NaCl	256.7					486
128.3 mmol/L NaCl	128.3					253
64.2 mmol/L NaCl	64.2					124
32.1 mmol/L NaCl	32.1					62
16.0 mmol/L NaCl	16					35
8.0 mmol/L NaCl	8					21
513.3 mmol/L NaCl + MMCK	513.3	27.4	25.2	9.9	10.7	1,092
513.3 mmol/L NaCl + K	513.3				10.7	971
513.3 mmol/L NaCl + MK	513.3		25.2		10.7	1,037
513.3 mmol/L NaCl + CK	513.3			9.9	10.7	973
513.3 mmol/L NaCl + MCK	513.3		25.2	9.9	10.7	1,062
17.1 mmol/L NaCl	17.1					31
17.1 mmol/L NaCl + 1.2 MCK	17.1		30.5	11.7	13.4	158
8.6 mmol/L NaCl	8.6					16
8.6 mmol/L NaCl + MCK	8.6		25.2	9.9	10.7	123
4.3 mmol/L NaCl	4.3					9
4.3 mmol/L NaCl + 1.2 MC	4.3		30.5	11.7		116
4.3 mmol/L NaCl + 1.2 K	4.3				13.4	33
4.3 mmol/L NaCl + 1.2 MCK	4.3		30.5	11.7	13.4	123
4.3 mmol/L NaCl + K	4.3				10.7	34
4.3 mmol/L NaCl + MCK	4.3		25.2	9.9	10.7	123
Natural seawater	N. D.	N. D.	N. D.	N. D.	N. D.	311

Note

Concentrations of dissolved salts and measured osmotic concentration are shown by mmol/L and mOsm/kg, respectively. 513.3 mmol/L NaCl corresponds to 3% NaCl. N. D. means not determined.

### 2.2 **Carbonic anhydrase and Na^+^**/**H^+^ exchanger inhibitors**

Acetazolamide and amiloride hydrochloride hydrate, which have already been used in crustacean studies (Burnett & Towle, 1990; Genovese et al., 2005; Henry et al., 2003; Tresguerres et al., 2008), were used as inhibitors for carbonic anhydrase (CA) and the NHE, respectively. Acetazolamide was dissolved in dimethyl sulfoxide (DMSO) to prepare a 200 mg/ml (1.11 mol/L) stock solution. In order to assess the effect of acetazolamide on survivals, four individuals of the crabs were reared in natural seawater containing 1 mg/ml (5.6 mmol/L) of acetazolamide 2 days prior to experiments and then transferred to the 513.3 mmol/L NaCl solution containing 1 mg/ml of acetazolamide. As the control group, the same number of the crabs were also reared in natural seawater and then transferred to the 513.3 mmol/L NaCl solution supplemented only with DMSO. Amiloride hydrochloride hydrate was dissolved in DMSO to prepare a 50 mg/ml (187.9 mmol/L) stock solution. In order to assess the effect of amiloride on survivals, four individuals of the crabs were reared in 513.3 mmol/L NaCl solution containing 0.25 mg/ml (0.9 mmol/L) of amiloride hydrochloride hydrate. The control group was reared in 513.3 mmol/L NaCl containing only DMSO. The survival rate was calculated 24 hr after transfer. The experiments were performed at least in triplicate.

### 2.3 **Hemolymph Na^+^**, **K^+^, and osmotic concentrations**

The crabs were anesthetized and dissected, and ~300 μl (for *H. tridens* and *C. dehaani*) or ~150 μl (for *M. japonicus*) of hemolymph was collected from each crab. They were centrifuged at 2,390 *g* for 5 min at 4°C and the supernatant was collected to be used as the sample solution. Na^+^ and K^+^ concentrations in 1:10 diluted and undiluted sample solutions, respectively, were measured using sodium (model C‐122) and potassium ion meters (model C‐131) (HORIBA, Kyoto, Japan), respectively. The osmotic concentrations were measured with a vapor pressure osmometer (Model 5600, Wescor, Logan, UT, USA).

### 2.4 **Amplification and isolation of Na^+^/K^+^ ATPase α subunit, cytoplasmic carbonic anhydrase, Na^+^/H^+^ exchanger, and Na^+^/K^+^/2Cl** ^−^ **cotransporter**

Amplification and isolation of complementary DNAs (cDNAs) encoding NKA α subunit, CAc, NHE, and NKCC in *H. tridens*, *M. japonicus*, and *C. dehaani* were performed, as previously described (Yamaguchi & Wakahara, 2001). Briefly, the most posterior pair of gills was isolated from crabs, and total RNA was extracted using Isogen reagent (Nippon Gene, Tokyo, Japan) according to the manufacturer's instructions. cDNAs were synthesized using an oligo (dT) primer and ReverTra Ace (TOYOBO, Osaka, Japan), and resultant cDNAs served as templates for polymerase chain reaction (PCR) amplification. Degenerate primers were designed on the basis of the amino acid sequence of the gene products in *Callinectes sapidus, Carcinus maenas*, *Portunus trituberculatus*, and *L. vannamei*. The primer sequences used for PCR were as follows: NKA α subunit: forward, 5′‐ATGACNGTNGCNCAYATGTGG‐3′; reverse, 5′‐GGRTGRTCNCCNGTNACCAT‐3′; CAc: forward, 5′‐TAYGTNTTRGARCARTTYCA‐3′ or 5′ ‐TAYGTNCTNGARCARTTYCA‐3′; reverse, 5′‐CKRAANGCRTCYAAYTG‐3' or 5′′‐CKRAANGCRTCNAGYTG‐3′; NHE: forward, 5′‐AARATHGGNTTYCAYATGAC‐3′; reverse, 5′‐TGYTCNACRTARTTYTTCAT‐3′; NKCC: forward, 5′‐AAYATHTGGGGNGTNATG‐3′; reverse, 5′‐GCRAARCANCCNGCRTADAT‐3′.

For amplification of CAc and NKCC cDNA, we performed nested PCR using part of the products from the first amplification as a template. The primer sequences used for nested PCR were as follows: CAc: forward, 5′‐CAYTGGGGNAARACNAAYGA‐3′; reverse, 5′‐CANGGNGGNGTNGTNA‐3′; NKCC: forward, 5′‐ATHTGGGGNGTNATGYT‐3′; reverse, 5′‐ARYTCCATNACYTGRAARCT‐3′.

In every case, conditions for PCR were as follows: 40 cycles for 30 s at 94°C, 30 s at annealing temperature, and 30 s at 72°C. The annealing temperature for NKA α subunit, CAc, NHE, and NKCC was 50°C, 43°C, 45°C, and 45°C, respectively. Amplified PCR products were electrophoresed and extracted, and then inserted into a pTA2 cloning vector (TOYOBO), and finally, their nucleotide sequences were determined. The sequences of isolated cDNAs were deposited to GenBank (Accession numbers of NKA α subunit, CAc, NHE, and NKCC are LC214855, LC375964, LC572286, and LC572287 for *H. tridens*, LC214856, LC375965, LC572288, and LC572289 for *M, japonicus*, and LC572290, LC572291, LC572292, and LC572293 for *C. dehaani*). In addition, amino acid sequences were predicted from the cDNA fragment sequences of *H. tridens*, *M. japonicus*, and *C. dehaani* and compared with those of the shore crab *C. maenas* (GenBank Accession numbers are AY03550, EU273943, U09274, and AY035548 for NKA α subunit, CAc, NHE, and NKCC, respectively) (Serrano & Henry, 2008; Towle et al., 1997).

### 2.5 Northern blotting

Northern blotting was performed, as previously described (Yamaguchi et al., 2000). Briefly, using cDNA fragments for genes encoding NKA α subunit, CAc, NHE, and NKCC as templates, digoxigenin‐labeled RNA probes were synthesized according to the manufacturer's instructions for the 10× DIG RNA labeling mix (Roche, Basel, Switzerland). Total RNA was extracted from the most posterior pair of gills using Isogen reagent (Nippon Gene), and 8.5μg of total RNA was electrophoresed in 1% agarose gel containing 1× 3‐(*N*‐morpholino)propanesulfonic acid (MOPS) and 5.5% formalin. Next, RNA was transferred from the gel to a positively charged nylon membrane (Roche) overnight. The resultant membrane was prehybridized using a solution containing 5× saline‐sodium citrate (SSC), 50% formamide, 5× Denhardt's solution, 0.5% sodium dodecyl sulfate (SDS), and 0.005% transfer RNA (tRNA) and then hybridized with digoxigenin‐labeled RNA probes overnight. Finally, the membrane was washed, blocked, and treated with alkaline phosphatase‐conjugated anti‐digoxigenin antibody (Roche) at a dilution of 1:2,000. Signals were detected using nitro blue tetrazolium and 5‐bromo‐4‐chloro‐3‐indolyl‐phosphate. The intensity of detected signals of Northern blotting was normalized with amount of electrophoresed ribosomal RNA, both of which were quantified using image processing program, ImageJ (National Institute of Health, Maryland, USA). The relative expression level was calculated as the expression in 513.3 mmol/L NaCl + MCK (Figure 5) or 4.3 mmol/L NaCl solution (Figure 9) was 1.0.

### 2.6 RNase protection assay

RNase protection assay was performed according to Sugiyama et al. (Sugiyama et al., 2001). Briefly, total RNA was extracted from the most posterior pair of gills and hybridized with digoxigenin‐labeled RNA probes overnight. Next, RNA was treated with RNase A and RNase T1 to digest single‐stranded RNA and electrophoresed in 1% agarose gel containing 1× MOPS and 5.5% formalin. Subsequently, RNA was transferred from the gel to a positively charged nylon membrane (Roche) overnight. Finally, the membrane was blocked and then treated with alkaline phosphatase‐conjugated anti‐digoxigenin antibody (Roche) at a dilution of 1:2,000. Signals were detected using nitro blue tetrazolium and 5‐bromo‐4‐chloro‐3‐indolyl‐phosphate. The intensity of the signals was quantified and the relative expression levels were determined as in Northern blotting.

### 2.7 Statistical analysis

All rearing experiments to evaluate survival rates were performed at least in triplicate, and Na^+^, K^+^, and osmotic concentrations were measured in at least three crabs in each group. Error bars in the figures show standard errors. Mean value comparisons among more than three groups were performed using analysis of variance (ANOVA), followed by Williams' test, Student's *t* test with Bonferroni correction, or Tukey's test. Mean value comparisons between two groups were performed by Student's *t* test. Results were considered statistically significant if the *p* value was <.05.

## 3 RESULTS

### 3.1 Effects of ambient minor cations on survival and hemolymph composition of euryhaline crabs under isosmotic conditions

**Figure 1:**
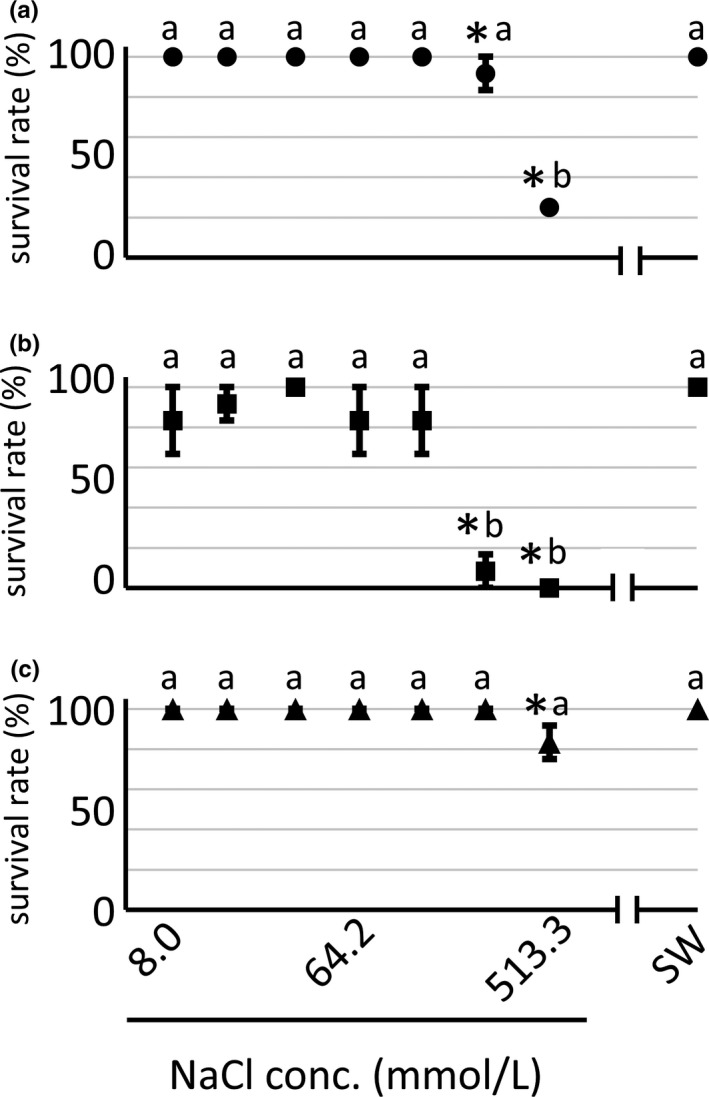
Survival rates of (a) *Helice tridens*, (b) *Macrophthalmus japonicus*, and (c) *Chiromantes dehaani* at various NaCl concentrations and in natural seawater 48 hr after initiation of rearing. The survival rate was determined in 8.0, 16.0, 32.1, 64.2, 128.3, 256.7, and 513.3 mmol/L NaCl solutions and seawater. Horizontal axes are scaled logarithmically. Values represent means of three experiments, and error bars represent standard errors. Significant differences were observed among different ambient NaCl concentrations in all three species (ANOVA, *p* < .05). *Significantly different values compared to seawater (*p* < .05; Williams’ test). Different letters indicate significantly different values among three species within the same NaCl concentration (*p* < .05; Tukey's test). SW, seawater

To investigate effect of minor cations on survivals of euryhaline crabs, *H. tridens*, *M. japonicus*, and *C. dehaani* were reared in 8.0, 16.0, 32.1, 64.2, 128.3, 256.7, and 513.3 mmol/L NaCl solution (i.e., 513.3 mmol/L NaCl solution was geometrically diluted with a factor of 2.0) and natural seawater. The survival rate of all of three species was 100% in natural seawater and relatively high in 8.0 mmol/L NaCl solution (Figure 1), indicating that all three species are strongly euryhaline and are hyperregulators in low salinity. However, the survival rate significantly decreased in *H. tridens* and *M. japonicus* at higher NaCl concentrations, and the majority of crabs (most of *H. tridens* and all of *M. japonicus)* died in 513.3 mmol/L NaCl solution, although its salinity was comparable to that of seawater (Figure 1a,b). The few *H. tridens* individuals who survived in 513.3 mmol/L NaCl solution were moribund. These results showed that 513.3 mmol/L NaCl solution without minor cations caused severe damage in crabs. Profit analysis revealed that median lethal concentrations (LC_50_) for *H. tridens* and *M. japonicus* were 431.57 ± 37.1 mmol/L and 164.37 ± 22.3 mmol/L, respectively. In contrast, *C. dehaani* had a high survival rate (>80%) even in 513.3 mmol/L NaCl solution (Figure 1c), although we observed a few signs of damages, such as slowed movements, indicating that *C. dehaani* responds to ambient solutions differently compared to *H. tridens* and *M. japonicus*. Because *C. dehaani* showed high survival rates even in high concentrations of NaCl solutions, Profit analysis could not determine LC_50_ in this species.

**Figure 2:**
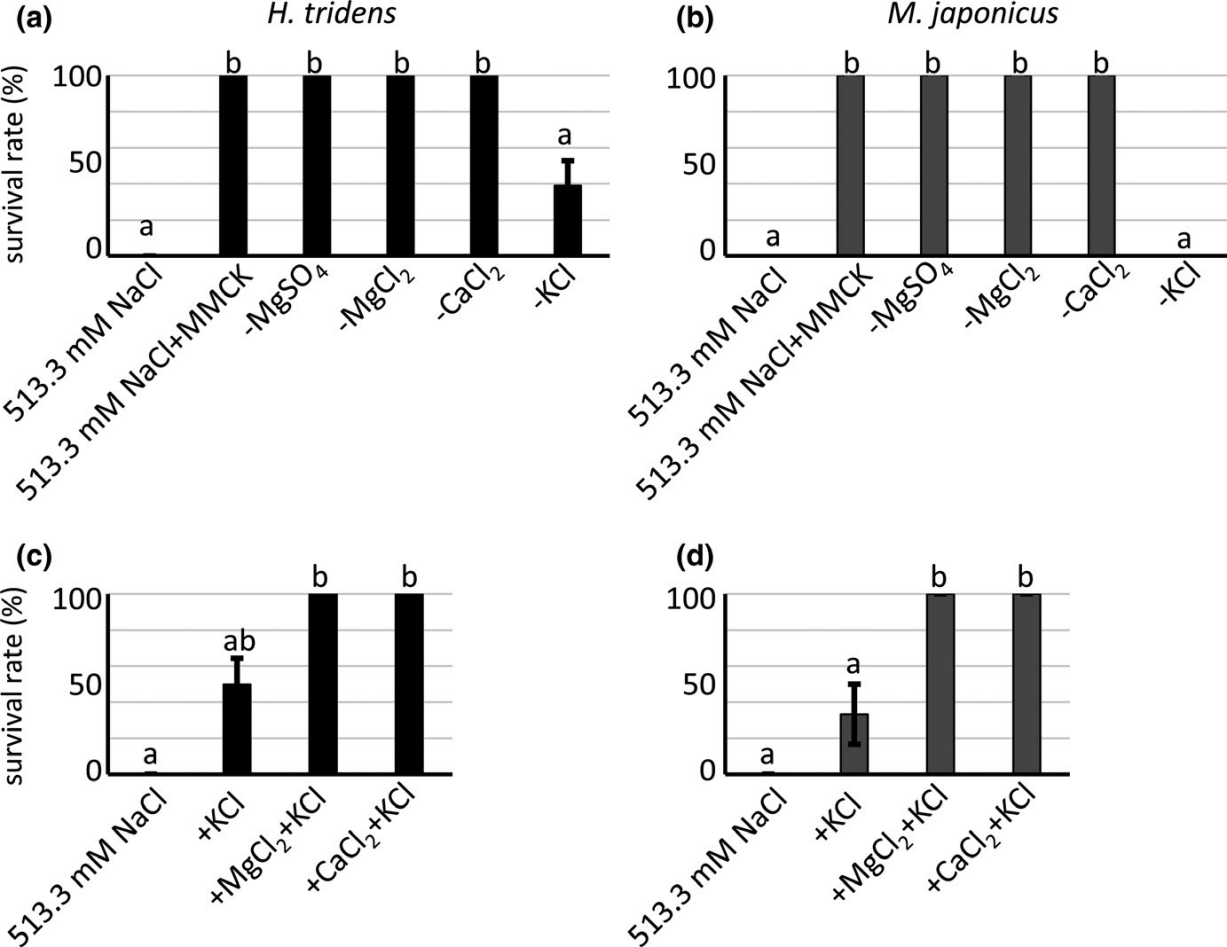
(a, b) Survival rates of (a) *Helice tridens* and (b) *Macrophthalmus japonicus* in 513.3 mmol/L NaCl solution, 513.3 mmol/L NaCl + MMCK solution, and 513.3 mmol/L NaCl + MMCK solution minus any one of the four additional salts. Values represent means of three experiments, and error bars represent standard errors. Significant differences were observed among different compositions of bathing media in both *H. tridens* and *M. japonicus* (ANOVA, *p* < .05). Different letters indicate significantly different values (*p* < .05; Tukey's test). (c, d) Survival rates of (c) *H. tridens* and (d) *M. japonicus* in 513.3 mmol/L NaCl, 513.3 mmol/L NaCl + K, and 513.3 mmol/L NaCl + K solutions supplemented with MgCl_2_ or CaCl_2_. Values represent means of three experiments, and error bars represent standard errors. Significant differences were observed among different compositions of bathing media in both *H. tridens* and *M. japonicus* (ANOVA, *p* < .05). Different letters indicate significantly different values (*p* < .05; Tukey's test). MMCK, 27.4 mmol/L MgSO4, 25.2 mmol/L MgCl_2_, 9.9 mmol/L CaCl_2_, and 10.7 mmol/L KCl; K, 10.7 mmol/L KCl

To determine whether administration of some ambient minor ions besides Na^+^ and Cl^−^ restore survival rate of *H. tridens* and *M. japonicus* in 513.3 mmol/L NaCl solution, 513.3 mmol/L NaCl solution was supplemented with 27.4 mmol/L MgSO_4_, 25.2 mmol/L MgCl_2_, 9.9 mmol/L CaCl_2_, and 10.7 mmol/L KCl (the resultant solution was referred to as 513.3 mmol/L NaCl + MMCK solution) to replicate the ionic composition of artificial seawater, and *H. tridens* and *M. japonicus* were reared in this solution. The survival rate significantly increased and reached 100% in both species (Figure 2a,b), indicating that 513.3 mmol/L NaCl + MMCK solution contains sufficient ambient minor ions for both species to survive in the presence of 513.3 mmol/L NaCl. To investigate which ambient minor ions are required for survival, *H. tridens* and *M. japonicus* were reared in several kinds of bathing media of isosmotic condition as follows; 513.3 mmol/L NaCl, 513.3 mmol/L NaCl + MMCK, and 513.3 mmol/L NaCl + MMCK minus any one of the four additional salts. The survival rate decreased in both species in the bathing medium lacking KCl (Figure 2a,b), indicating that K^+^ is indispensable for survival of both species in the presence of 513.3 mmol/L NaCl. To determine the specific role of K^+^, the 513.3 mmol/L NaCl solution was supplemented only with 10.7 mmol/L KCl (referred to as the 513.3 mmol/L NaCl + K solution), and *H. tridens* and *M. japonicus* were reared in this solution. The survival rate did not fully recover in both species in 513.3 mmol/L NaCl + K solution (Figure 2c,d), suggesting that addition of K^+^ alone is insufficient. Thus the 513.3 mmol/L NaCl + K solution was supplemented with either 25.5 mmol/L MgCl_2_ or 9.9 mmol/L CaCl_2_ and *H. tridens* and *M. japonicus* were reared in these solutions. Both 513.3 mmol/L NaCl + K + 25.5 mmol/L MgCl_2_ and 513.3 mmol/L NaCl + K + 9.9 mmol/L CaCl_2_ restored survival rate of both species (Figure 2c,d), indicating that the presence of both K^+^ and a divalent cation (either Mg^2+^ or Ca^2+^) is necessary and sufficient for the survival of *H. tridens* and *M. japonicus*.

**Figure 3:**
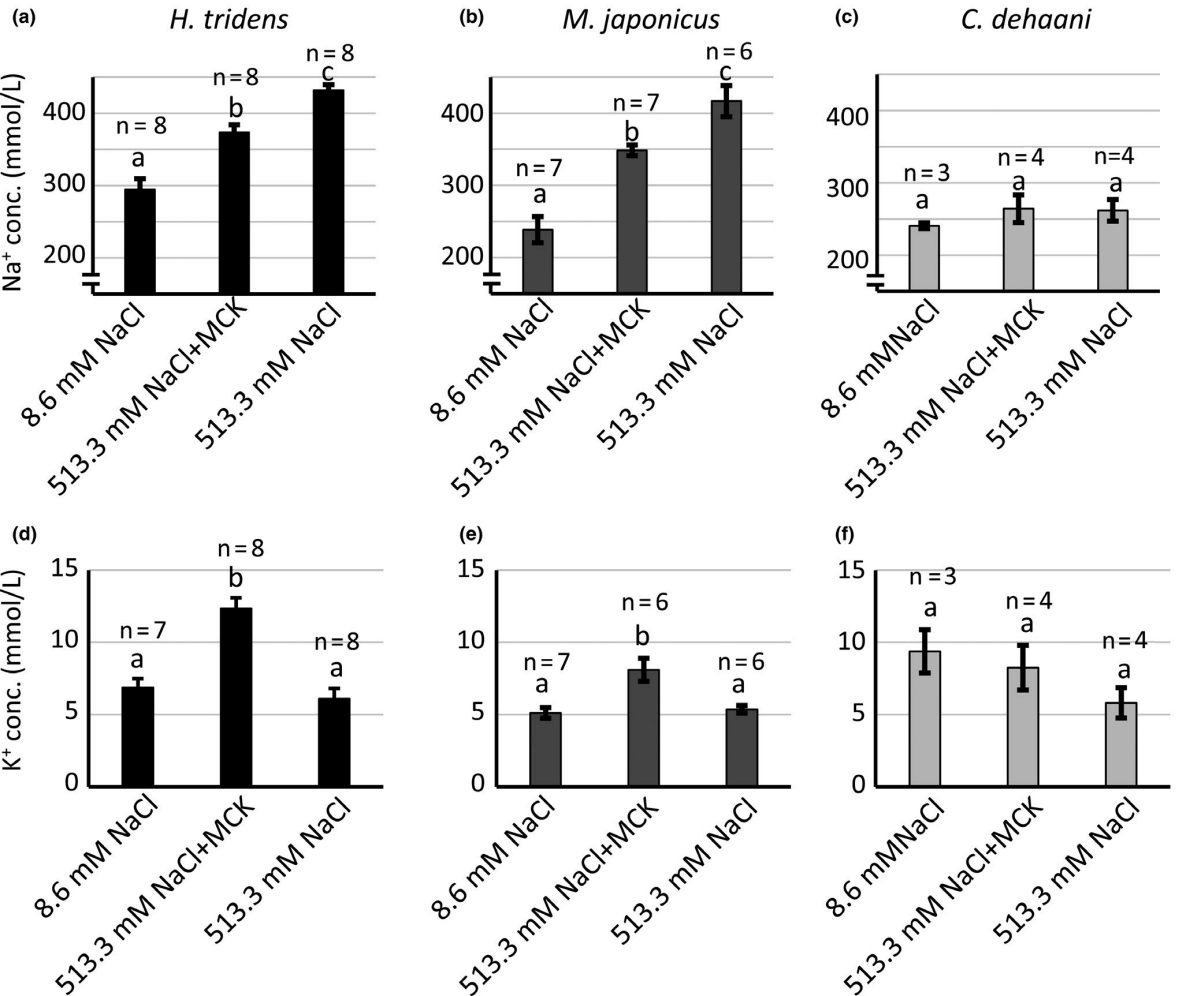
Hemolymph Na^+^ and K^+^ concentrations in (a, d) *Helice tridens*, (b, e) *Macrophthalmus japonicus*, and (c, f) *Chiromantes dehaani* 6 hr after initiation of incubation. Hemolymph (a–c) Na^+^ and (d–f) K^+^ concentrations in crabs held in 8.6 mmol/L NaCl, 513.3 mmol/L NaCl + MCK, and 513.3 mmol/L NaCl solution. Values represent means (number of analyzed crabs shown above each bar), and error bars represent standard errors. Significant differences of both Na^+^ and K^+^ concentrations were observed among different compositions of bathing media in *H. tridens* and *M. japonicus* (ANOVA, *p* < .05), but not in *C. dehaani* (ANOVA, *p* > .05). Different letters indicate significantly different values (*p* < .05; Student's *t* test with Bonferroni correction). MCK, 25.2 mmol/L MgCl_2_, 9.9 mmol/L CaCl_2_, and 10.7 mmol/L KCl

Next step of the study was to examine the hemolymph ionic composition and osmotic concentration of these crabs, *H. tridens*, *M. japonicus*, and *C. dehaani* incubated in the 8.6 mmol/L NaCl, 513.3 mmol/L NaCl supplemented with 25.5 mmol/L MgCl_2_, 9.9 mmol/L CaCl_2_, and 10.7 mmol/L KCl (referred to as 513.3 mmol/L NaCl + MCK solution), and 513.3 mmol/L NaCl solution. Hemolymph Na^+^, K^+^, and osmotic concentrations were determined at 6 hr after incubation. The hemolymph Na^+^ concentration in both *H. tridens* and *M. japonicus* held in 513.3 mmol/L NaCl solution was significantly higher compared to 513.3 mmol/L NaCl + MCK solution (Figure 3a,b). In addition, the hemolymph K^+^ concentration in crabs held in 513.3 mmol/L NaCl solution was significantly less compared to 513.3 mmol/L NaCl + MCK solution and was comparable to the hemolymph K^+^ concentration in crabs held in 8.6 mmol/L NaCl solution (Figure 3d,e). Moreover, hemolymph osmotic concentration in *H. tridens* held in 513.3 mmol/L NaCl solution was significantly higher compared to that in 513.3 mmol/L NaCl + MCK solution, but not in *M. japonicus* (Table 2). In contrast, differences in ionic compositions of bathing media did not change hemolymph Na^+^, K^+^, and osmotic concentrations in *C. dehaani*, which had a high survival rate in 513.3 mmol/L NaCl solution (Figure 3c,f; Table 2).

**Table 2:** Osmotic concentrations (mOsm/kg) of hemolymph bathed in 8.6 mmol/L NaCl, 513.3 mmol/L NaCl + MCK, and 513.3 mmol/L NaCl solutions

Treatment	*H. tridens*	*M. japonicus*	*C. dehaani*
8.6 mmol/L NaCl	710.0 ± 4.2^a^	660.3 ± 9.0^a^	518.0 ± 80.7^a^
513.3 mmol/L NaCl + MCK	771.3 ± 12.8^b^	894.0 ± 31.2^b^	703.7 ± 89.8^a^
513.3 mmol/L NaCl	937.0 ± 11.1^c^	907.7 ± 34.2^b^	602.0 ± 29.5^a^

Note

Values represent means (*n* = 3) ± standard errors. Significant differences were observed among different bathing media in *Helice tridens* and *Macrophthalmus japonicus* (ANOVA, *p* < .05), but not observed in *Chiromantes dehaani* (ANOVA, *p* > .05). Different letters indicate significantly different values within each species (*p* < .05; Student's *t* test with Bonferroni correction).

### 3.2 **Isolation and expression analysis of genes encoding Na^+^/K^+^ ATPase** α **subunit, cytoplasmic carbonic anhydrase, and Na^+^/H^+^ exchanger**

**Figure 4:**
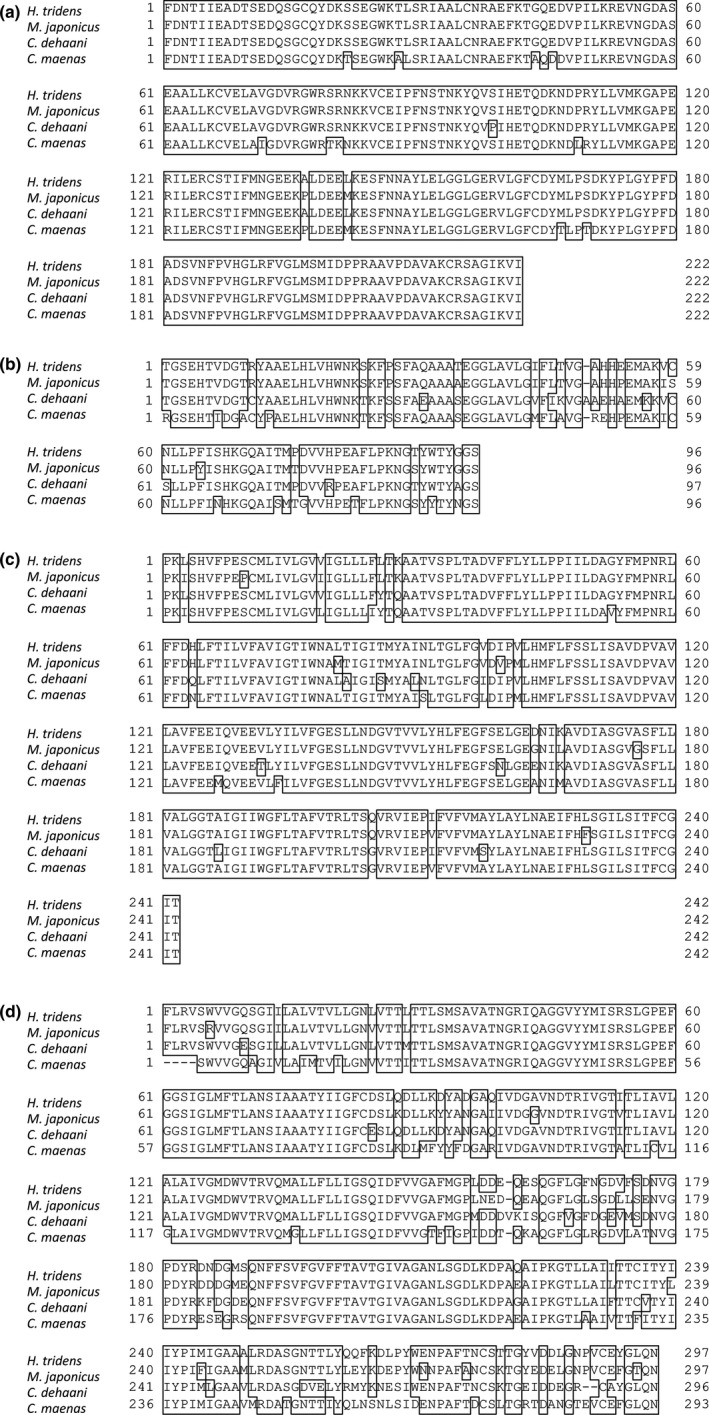
Amino acid sequences of (a) NKA α subunit, (b) CAc, (c) NHE, and (d) NKCC of *Helice tridens*, *Macrophthalmus japonicus*, and *Chiromantes dehaani* deduced from cDNA fragments and alignments with those of the shore crab *Carcinus maenas*

To analyze the possibility that changes in the expression of genes involved in Na^+^ uptake disturbed hemolymph ionic composition and ultimately led to the death of crabs in rearing experiments, cDNA fragments of NKA α subunit, CAc, and NHE were amplified by PCR and isolated, and gene expression was analyzed in the most posterior gills of *H. tridens*, *M. japonicus*, and *C. dehaani*. The lengths of isolated cDNA fragments for the NKA α subunit, CAc, and NHE genes were 700, 300, and 800 bp, respectively. Sequencing results showed that the cDNA fragment sequences of these genes were highly similar to those in other crab species. The deduced amino acid sequences of the NKA α subunit of *H. tridens*, *M. japonicus*, and *C. dehaani* had 95%, 95%, and 94% similarity, respectively, to that of *C. maenas*; those of CAc of *H. tridens*, *M. japonicus*, and *C. dehaani* had 76%, 78%, and 75% similarity, respectively, to that of *C. maenas;* and those of the NHE of *H. tridens*, *M. japonicus*, and *C. dehaani* had 94%, 93%, and 92% similarity, respectively, to that of *C. maenas* (Figure 4a–c) (Serrano & Henry, 2008; Towle et al., 1997).

**Figure 5:**
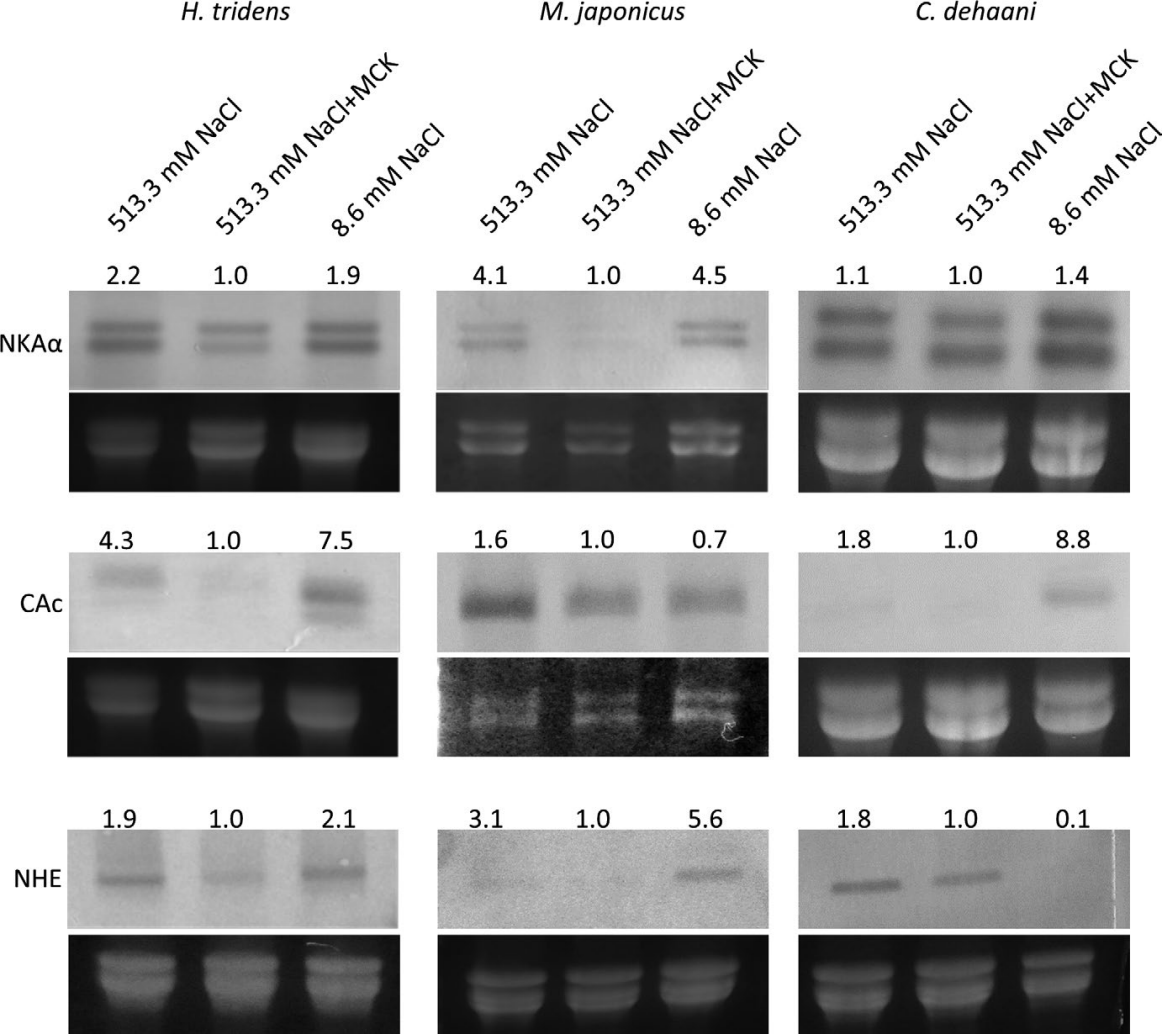
Expression of genes encoding NKA α subunit, CAc, and NHE in the most posterior gills of *Helice tridens*, *Macrophthalmus japonicus*, and *Chiromantes dehaani* 6 hr after initiation of incubation in 513.3 mmol/L NaCl, 513.3 mmol/L NaCl + MCK, and 8.6 mmol/L NaCl solutions. All gene expression was detected using Northern blotting, except the expression of CAc in *M. japonicus*, which was detected using RNase protection assay. The relative expression level was shown in each band as the expression in 513.3 mmol/L NaCl + MCK solution is 1.0. NKA, Na^+^/K^+^ ATPase; CAc, cytoplasmic carbonic anhydrase; NHE, Na^+^/H^+^ exchanger; MCK, 25.5 mmol/L MgCl_2_, 9.9 mmol/L CaCl_2_, and 10.7 mmol/L KCl

The expression of these genes in the most posterior gills of crabs held in the 8.6 mmol/L NaCl, 513.3 mmol/L NaCl + MCK, and 513.3 mmol/L NaCl solutions for 6 hr were analyzed by Northern blotting. However, the expression levels of CAc gene were not sufficiently high in *M. japonicus* to be detected by Northern blotting; therefore, RNase protection assay was carried out to detect the expression of this gene in *M. japonicus*. These analyses revealed that the expression of all genes in the most posterior gills in *H. tridens* and *M. japonicas* was higher in 8.6 mmol/L NaCl solution compared to 513.3 mmol/L NaCl + MCK solution except CAc in *M. japonicus*. Furthermore, the expression of all genes in the most posterior gills was also higher in 513.3 mmol/L NaCl solution compared to 513.3 mmol/L NaCl + MCK solution as in 8.6 mmol/L NaCl solution (Figure 5). On the other hand, the expression of these genes was different in *C. dehaani*. The expression of genes encoding NKA α subunit was enhanced in 513.3 mmol/L NaCl solution but was less drastic compared to *H. tridens* and *M. japonicus*. In addition, the expression of CAc gene was not induced in 513.3 mmol/L NaCl solution but was in 8.6 mmol/L NaCl solution. In contrast, the expression of NHE gene was enhanced in 513.3 mmol/L NaCl solution but not in 8.6 mmol/L NaCl solution (Figure 5).

### 3.3 Effects of carbonic anhydrase and Na^+^/H^+^ exchanger inhibitors

**Figure 6:**
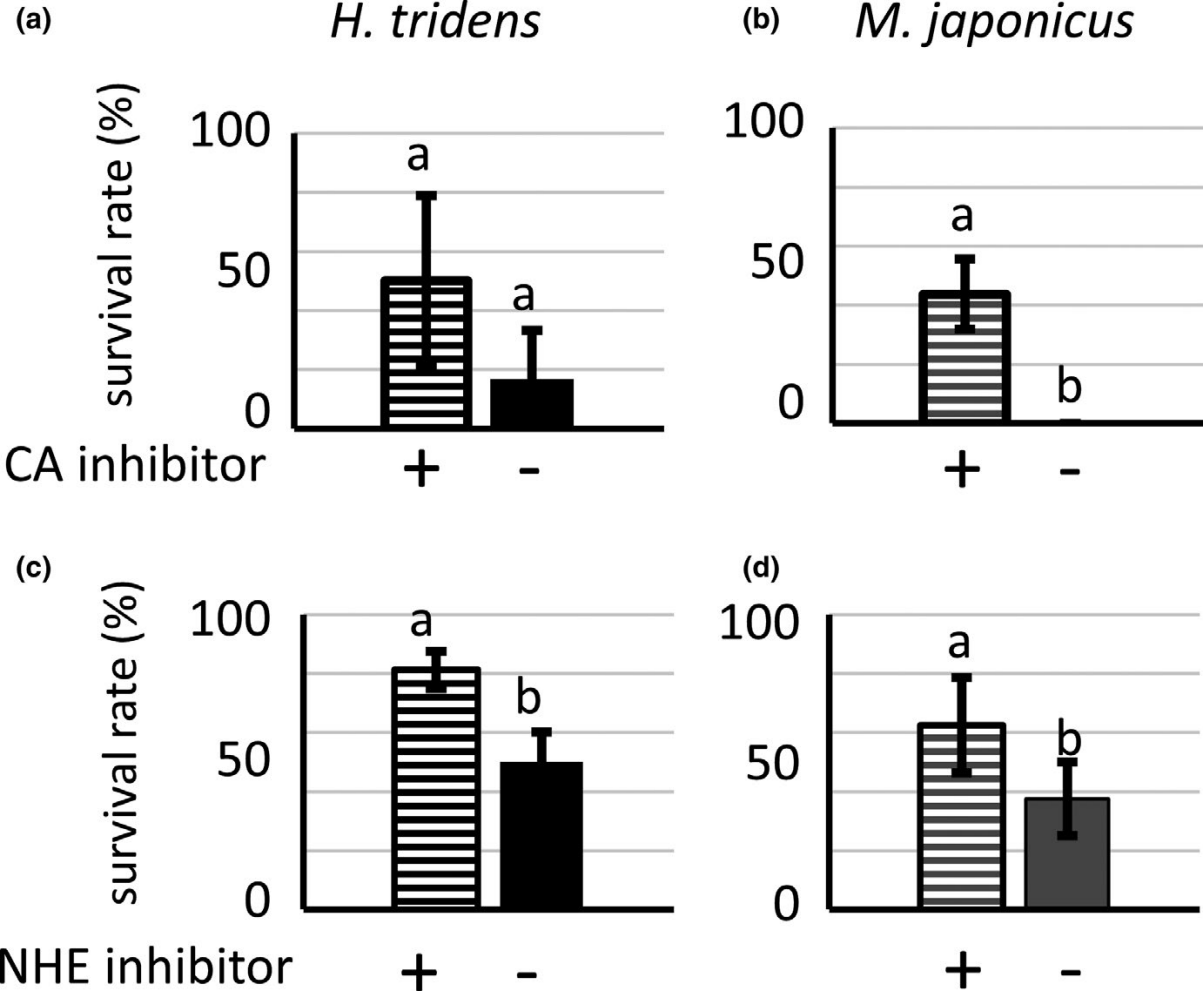
Survival rates of (a, c) *Helice tridens* and (b, d) *Macrophthalmus japonicus* in 513.3 mmol/L NaCl solution with or without acetazolamide, an inhibitor of CA (a and b), or amiloride hydrochloride hydrate, an inhibitor of NHE (c and d), 24 hr after initiation of rearing. Values represent means of three (for *H. tridens*) or four (for *M. japonicus*) experiments, and error bars represent standard errors. Different letters indicate significantly different values (*p* < .05; Student's *t* test). CA, carbonic anhydrase; NHE, Na^+^/H^+^ exchanger

Increased expression of genes involved in Na^+^ uptake could be a possible cause of the death of *H. tridens* and *M. japonicus* in 513.3 mmol/L NaCl solution. To examine this possibility, *H. tridens* and *M. japonicas* were reared in 513.3 mmol/L NaCl solution supplemented with acetazolamide or amiloride, an inhibitor of CA and NHE, respectively. Amiloride significantly increased the survival rate of *H. tridens* and *M. japonicus*, and acetazolamide increased the survival rate of *M. japonicus* reared in 513.3 mmol/L NaCl solution (Figure 6b–d). The survival rate was also higher in *H. tridens* reared in 513.3 mmol/L NaCl solution supplemented with acetazolamide compared to 513.3 mmol/L NaCl solution, but the result was not statistically significant (Figure 6a).

### 3.4 Effects of ambient minor cations on survival and hemolymph composition of euryhaline crabs under hypo‐osmotic conditions

**Figure 7:**
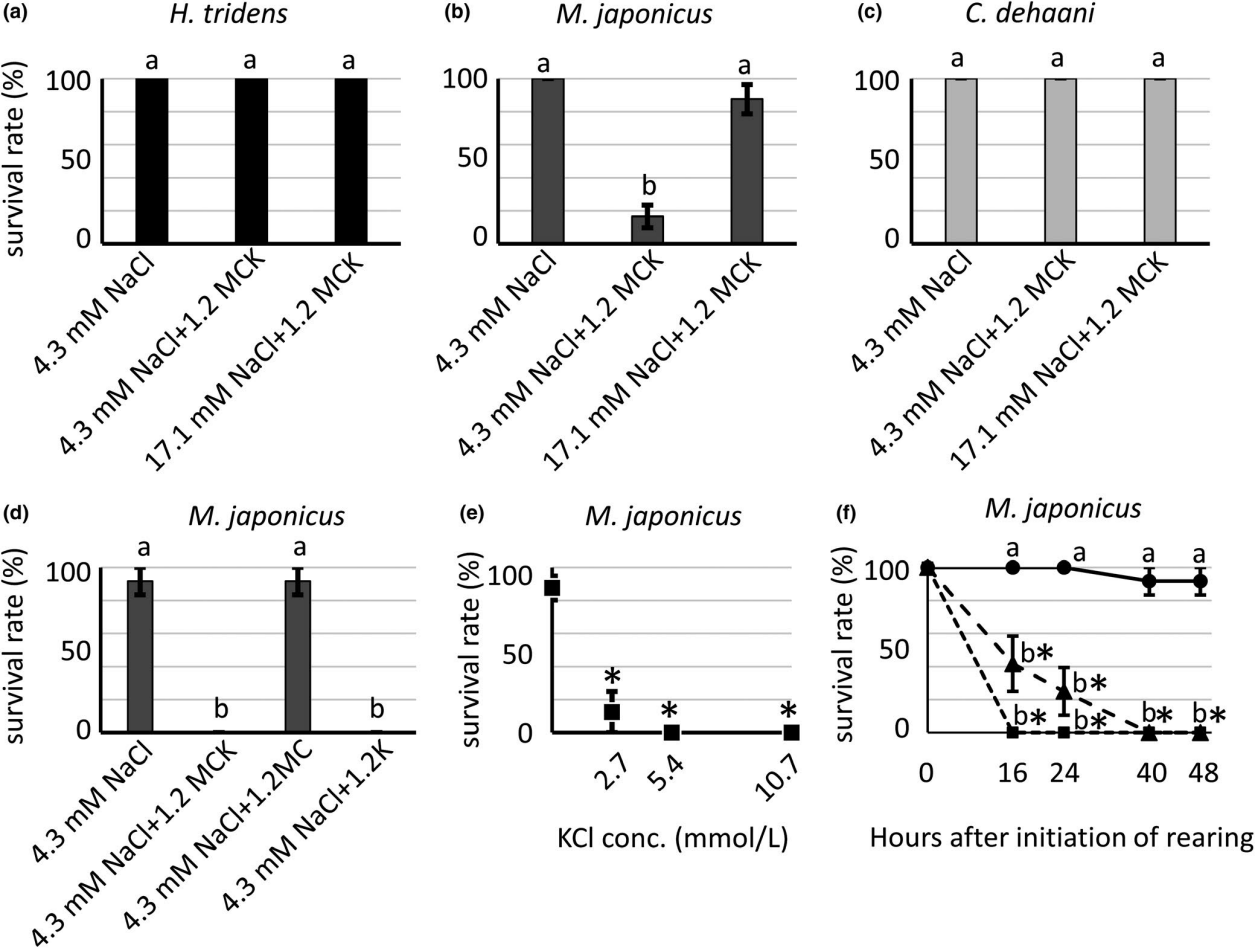
Survival rates of (a) *Helice tridens*, (b) *Macrophthalmus japonicus*, and (c) *Chiromantes dehaani* reared in 4.3 mmol/L NaCl, 4.3 mmol/L NaCl + 1.2 MCK, and 17.1 mmol/L NaCl + 1.2 MCK solutions. Values represent means of four (for *H. tridens* and *M. japonicas*) or three (for *C. dehaani*) experiments, and error bars represent standard errors. Significant differences were observed among different compositions of bathing media in *M. japonicus* (ANOVA, *p* < .05), but not in *H. tridens* and *C. dehaani* (ANOVA, *p* > .05). Different letters indicate significantly different values (*p* < .05; Student's *t* test with Bonferroni correction). (d) Survival rate of *M. japonicus* in 4.3 mmol/L NaCl, 4.3 mmol/L NaCl + 1.2 MCK, and 4.3 mmol/L NaCl solutions supplemented with 30.5 mmol/L MgCl_2_ and 11.7 mmol/L CaCl_2_ (1.2 MC) or 13.4 mmol/L KCl (1.2 K). Values represent means of three experiments, and error bars represent standard errors. Significant differences were observed among different compositions of bathing media (ANOVA, *p* < .05). Different letters indicate significantly different values (*p* < .05; Tukey's test). (e) Survival rate of *M. japonicus* in 4.3 mmol/L NaCl solution supplemented with 0, 2.7, 5.4, and 10.7 mmol/L KCl 24 hr after initiation of rearing. Values represent means of four experiments, and error bars represent standard errors. *Significantly different values from that in 4.3 mmol/L NaCl solution without KCl. (*p* < .05; Williams' test). (f) Survival rate of *M. japonicus* reared in 4.3 mmol/L NaCl (circles), 4.3 mmol/L NaCl + 1.2 MCK (triangles), and 4.3 mmol/L NaCl + 1.2 K (squares) solutions 15, 24, 39, and 48 hr after initiation of experiments. Values represent means of three experiments, and error bars represent standard errors. Different letters indicate significantly different values in each time point (*p* < .05; Tukey's test). *Significantly different values from the beginning point (*p* < .05; Williams' test). 1.2 MCK, 30.5 mmol/L MgCl_2_, 11.7 mmol/L CaCl_2_, and 13.4 mmol/L KCl; 1.2 MC, 30.5 mmol/L MgCl_2_ and 11.7 mmol/L CaCl_2_; 1.2 K, 13.4 mmol/L KCl

To investigate the effects of ambient minor cations on the survival of euryhaline crabs at low ambient salinity, *H. tridens*, *M. japonicus*, and *C. dehaani* were reared in 8.6 mmol/L NaCl solution supplemented with 25.2 mmol/L MgCl_2_, 9.9 mmol/L CaCl_2_, and 10.7 mmol/L KCl (referred to as 8.6 mmol/L NaCl + MCK solution), 8.6 mmol/L NaCl solution, and 17.1 mmol/L NaCl solution without additional salts. Additional salts did not have any effect on the survival of *H. tridens* and *C. dehaani*, showing 100% survival in both species (data not shown). In contrast, the survival rate considerably decreased in *M. japonicus* in 8.6 mmol/L NaCl + MCK solution (data not shown). Because some *M. japonicus* individuals still appeared intact in 8.6 mmol/L NaCl + MCK solution, further experiments were conducted using 4.3 mmol/L NaCl, 4.3 mmol/L NaCl supplemented with 30.5 mmol/L MgCl_2_, 11.7 mmol/L CaCl_2_, and 13.4 mmol/L KCl (referred to as 4.3 mmol/L NaCl + 1.2 MCK solution), and 17.1 mmol/L NaCl + 1.2 MCK solution. The survival rate of *H. tridens* and *C. dehaani* was 100%, but that of *M. japonicus* significantly decreased to 17% in 4.3 mmol/L NaCl + 1.2 MCK solution (Figure 7). A few *M. japonicus* individuals survived in this solution but were moribund. In contrast, most *M. japonicus* individuals survived in 4.3 mmol/L NaCl and 17.1 mmol/L NaCl + 1.2 MCK solution (Figure 7b). These results indicated that higher concentrations of additional salts in combination with a lower NaCl concentration caused high mortality in *M. japonicus* in 4.3 mmol/L NaCl + 1.2 MCK solution.

To examine which minor cations have lethal effect on *M. japonicus*, 30.5 mmol/L MgCl_2_ and 11.7 mmol/L CaCl_2_ (1.2MC) and 13.4 mmol/L KCl (1.2K) were added to 4.3 mmol/L NaCl solution separately. In *M. japonicus* reared in 4.3 mmol/L NaCl solution supplemented with 13.4 mmol/L KCl (referred to as 4.3 mmol/L NaCl + 1.2 K solution), the survival rate significantly decreased to a comparable level as that in 4.3 mmol/L NaCl + 1.2 MCK solution (Figure 7d). Even after diluting KCl to 2.7 mmol/L (almost one‐fourth of the concentration in seawater), the survival rate hardly recovered (Figure 7e), indicating that K^+^ accounts for the lethality in 4.3 mmol/L NaCl + 1.2 MCK solution. In contrast, supplementation of 4.3 mmol/L NaCl with 30.5 mmol/L MgCl_2_ and 11.7 mmol/L CaCl_2_ did not have a lethal effect on *M. japonicus*; instead, these divalent cations attenuated the lethal effect of K^+^ 15 hr after initiation of rearing, although it did not block the lethal effect at all 48 hr after initiation of rearing (Figure 7d,f).

**Figure 8:**
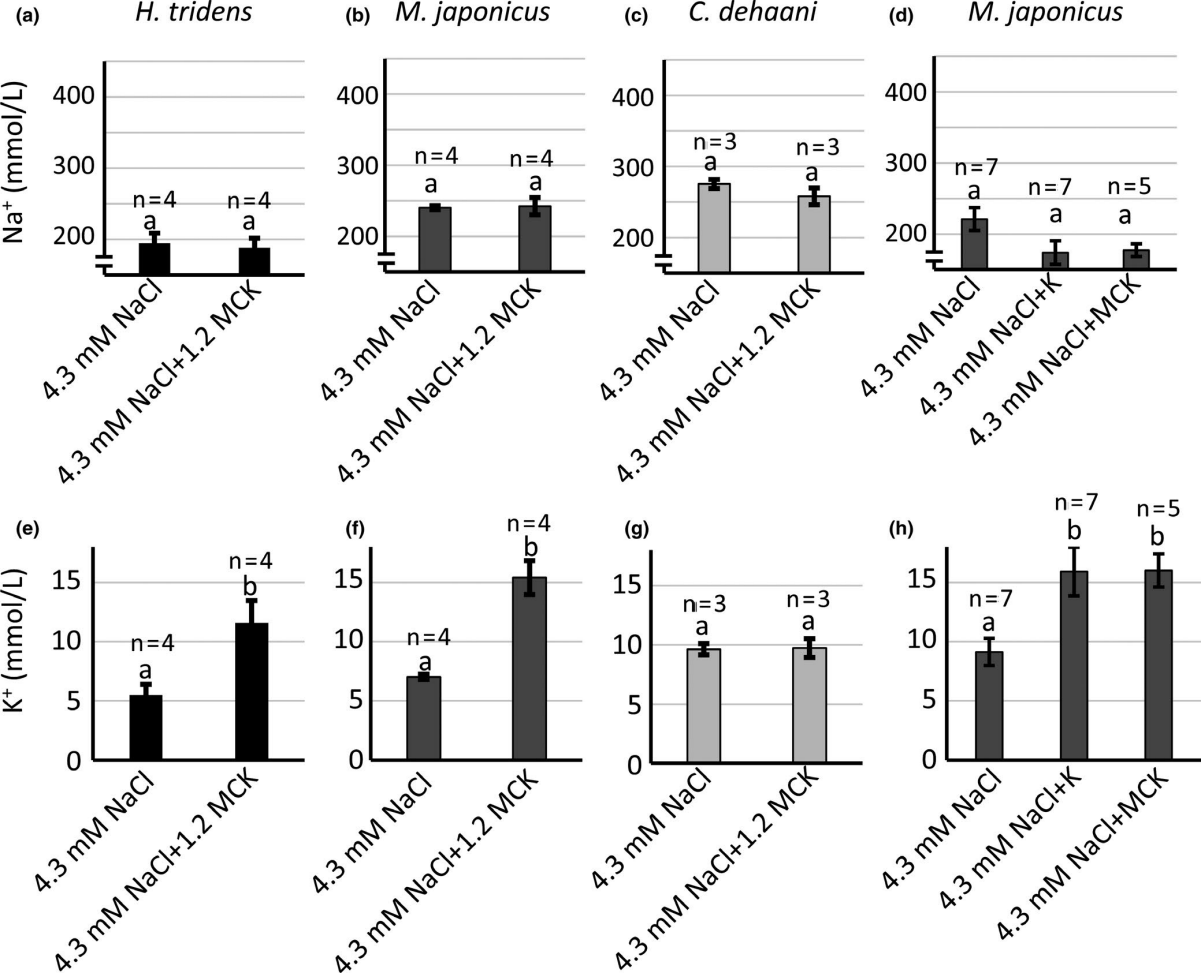
Hemolymph (a–d) Na^+^ and (e–h) K^+^ concentrations in (a, e) *Helice tridens*, (b, d, f, h) *Macrophthalmus japonicus*, and (c, g) *Chiromantes dehaani* incubated in (a–c, e–g) 4.3 mmol/L NaCl and 4.3 mmol/L NaCl + 1.2 MCK solutions or in (d, h) 4.3 mmol/L NaCl, 4.3 mmol/L NaCl + K, and 4.3 mmol/L NaCl + MCK solutions 6 hr after initiation of incubation. Values represent means (number of analyzed crabs shown above each bar), and error bars represent standard errors. Significant differences were observed among different compositions of bathing media in (h) (ANOVA, *p* < .05), but not in (d) (ANOVA, *p* > .05). Different letters indicate significantly different values (*p* < .05; Student's *t* test for a–c and e–g, and Student's *t* test with Bonferroni correction for d and h). 1.2 MCK, 30.5 mmol/L MgCl_2_, 11.7 mmol/L CaCl_2_, and 13.4 mmol/L KCl; MCK, 25.2 mmol/L MgCl_2_, 9.9 mmol/L CaCl_2_, and 10.7 mmol/L KCl; K, 10.7 mmol/L KCl

Possible effects of ambient minor cations under hypo‐osmotic conditions on hemolymph ionic composition and osmotic concentration were examined in *H. tridens*, *M. japonicus*, and *C. dehaani*. They were incubated in the 4.3 mmol/L NaCl and 4.3 mmol/L NaCl + 1.2 MCK solutions, and hemolymph Na^+^, K^+^, and osmotic concentrations were determined 6 hr after incubation. There were no significant differences in the hemolymph Na^+^ concentration between crabs incubated in 4.3 mmol/L NaCl and 4.3 mmol/L NaCl + 1.2 MCK solutions in all three species (Figure 8a–c). In contrast, the hemolymph K^+^ concentration in crabs incubated in 4.3 mmol/L NaCl + 1.2 MCK solution was significantly higher compared to 4.3 mmol/L NaCl solution in *H. tridens* and *M. japonicus* but not in *C. dehaani* (Figure 8e–g). Moreover, addition of minor cations increased osmotic concentration significantly in *M. japonicus*, but not in *H. tridens* and *M. japonicus* (Table 3). Additional experiments were conducted to know specific role of K^+^ on the hemolymph ionic composition in *M. japonicus*. Administration of KCl increased the hemolymph K^+^ concentration as combined administration of MgCl_2_, CaCl_2_, and KCl (Figure 8h).

**Table 3:** Osmotic concentrations (mOsm/kg) of hemolymph bathed in 4.3 mmol/L NaCl and 4.3 mmol/L NaCl + 1.2 MCK solutions

Treatment	*H. tridens*	*M. japonicus*	*C. dehaani*
4.3 mmol/L NaCl	680.7 ± 10.2^a^	545.3 ± 8.5^a^	649.0 ± 9.0^a^
4.3 mmol/L NaCl + 1.2 MCK	642.3 ± 28.2^a^	618.7 ± 13.9^b^	635.0 ± 12.7^a^

Note

Values represent means (*n* = 3) ± standard errors. Different letters indicate significantly different values within each species (*p* < .05; Student's *t* test).

### 3.5 **Isolation of the gene encoding Na^+^/K^+^/2Cl** ^−^ **cotransporter and expression analysis of the gene and Na^+^/K^+^ ATPase** α **subunit under hypo‐osmotic conditions**

**Figure 9:**
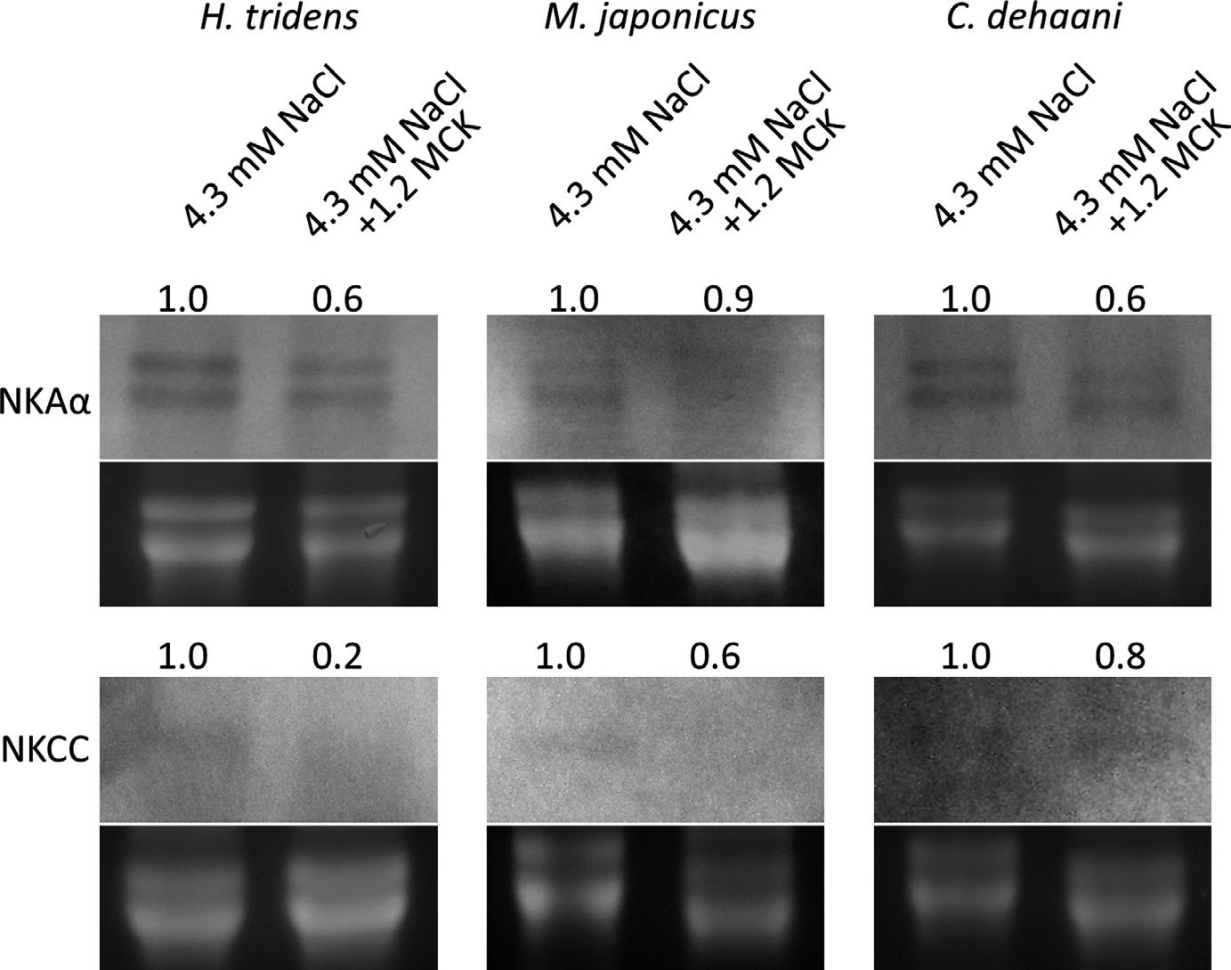
Expression of genes encoding NKA α subunit and NKCC in the most posterior gills of *Helice tridens*, *Macrophthalmus japonicus*, and *Chiromantes dehaani* 6 hr after initiation of incubation in 4.3 mmol/L NaCl and 4.3 mmol/L NaCl + 1.2 MCK solutions. All expressions were detected using Northern blotting. The relative expression level was shown in each band as the expression in 4.3 mmol/L NaCl solution is 1.0. NKA, Na^+^/K^+^ ATPase; NKCC, Na^+^/K^+^/2Cl^−^ cotransporter; 1.2 MCK, 30.5 mmol/L MgCl_2_, 11.7 mmol/L CaCl_2_, and 13.4 mmol/L KCl

To analyze the possibility that changes in the expression of genes involved in K^+^ transport disturbed hemolymph K^+^ concentration and ultimately led to the death of *M. japonicus* in 4.3 mmol/L NaCl + 1.2 MCK solution, cDNA fragment of NKCC was amplified by PCR and isolated, and gene expression of it and NKA α subunit was analyzed in the most posterior gills of *H. tridens*, *M. japonicus*, and *C. dehaani*. The deduced amino acid sequence of NKCC of *H. tridens*, *M. japonicus*, and *C. dehaani* had 81%, 79%, and 77% similarity to that of *C. maenas* (Figure 4d). Northern blotting revealed that NKA α subunit expression in the most posterior gills of crabs incubated in 4.3 mmol/L NaCl + 1.2 MCK solution for 6 hr decreased compared to 4.3 mmol/L NaCl solution in all three species (Figure 9). In *H. tridens* and *M. japonicus*, NKCC expression also decreased in the presence of ambient minor cations, although the expression was relatively low (Figure 9). In contrast, NKCC expression in *C. dehaani* was less affected by ambient minor cations (Figure 9).

## 4 DISCUSSION

In this study, three species of euryhaline crabs, *H. tridens*, *M. japonicus*, and *C. dehaani* were used. Habitats of *H. tridens* and *M. japonicus* are restricted within estuaries, but *C. dehaani* is a species which invaded more upstream regions and is adaptive to fresh water (Irawan & Kijima, 1993; Kobayashi, 2000). These species belong to three different families: Varunidae (*H. tridens*), Ocypodidae (*M. japonicus*), and Sesarmidae (*C. dehaani*), although *H. tridens* and *C. dehaani* belong to the same superfamily, Grapsoidea. The different responses to environmental water of these species are possibly due to the differences in the microenvironment of their habitats or phylogeny.

### 4.1 Effect of ambient minor cations in seawater on the survival of euryhaline crabs

It has been reported that not only salinity but also ionic composition of the environment significantly affects the survival of euryhaline crustaceans. For example, the mortality of the euryhaline prawn *P. mondon* reaches 100% during the first 48 hr in 0.17% NaCl solution, although it remains low in diluted artificial seawater of the same salinity (Cawthorne et al., 1983), indicating that some ambient minor ions, besides Na^+^ and Cl^−^, are essential for its survival. In addition, analysis of ionic profiles of inland well water for cultivation of prawns shows that the presence of K^+^, Mg^2+^, and SO_4_^2‐^ increases the survival rate of euryhaline prawns *L. vannamei* and *M. latisulcatus* (Davis et al., 2005; Prangnell & Fotedar, 2005, 2006; Roy et al., 2007; Saoud et al., 2003). Furthermore, removal of either Ca^2+^ or Mg^2+^ or both, but not K^+^, from seawater causes lethal damage to the shore crab *C. maenas*, showing that both divalent cations, but not K^+^, are required for its survival (Lovett et al., 2006).

This study revealed that both *H. tridens* and *M. japonicus* can survive even in 8.6 mmol/L NaCl solution but not in 513.3 mmol/L NaCl solution (Figure 1). The high mortality of *H. tridens* and *M. japonicus* in 513.3 mmol/L NaCl solution indicates some ambient minor ions besides Na^+^ and Cl^−^ are required for these crabs to survive under isosmotic condition. For complete recovery of the survival rate in 513.3 mmol/L NaCl solution, both K^+^ and divalent cations, either Mg^2+^ or Ca^2+^, are required (Figure 2). In addition, the required ambient minor ions for both *H. tridens* and *M. japonicus* are different compared to other euryhaline crustaceans previously examined: (1) SO_4_^2‐^ is not needed for survival of *H. tridens* and *M. japonicus* unlike *L. vannamei* (Saoud et al., 2003), (2) K^+^ and either Mg^2+^ or Ca^2+^ are essential for both *H. tridens* and *M. japonicus*, although both Mg^2+^ and Ca^2+^, but not K^+^, are required for *C. maenas* (Lovett, et al., 2006), and (3) while *P. mondon* cannot survive 48 hr in 0.17% NaCl solution (Cawthorne et al., 1983), both *H. tridens* and *M. japonicus* can survive in NaCl solution with similar salinity (32.1 mmol/L, corresponding to 0.19%). These data show that the required minor ions for survival differ among species of euryhaline crustaceans, and the osmotic condition itself in which the ambient minor ions are functioning also differ, respectively. This difference might, in turn, reflect the difference between the microenvironments of their habitats and/or phylogeny. On the other hand, *C. dehaani* has a significantly high survival rate, even in 513.3 mmol/L NaCl solution, in striking contrast to *H. tridens* and *M. japonicas* (Figure 1)*. Chiromantes dehaani* is more adaptive to lower salinity compared to *H. tridens* and *M. japonicus* (Irawan & Kijima, 1993), which would account for the robustness of this species in 513.3 mmol/L NaCl solution.

### 4.2 Role of ambient minor cations under isosmotic conditions with seawater

Under hypo‐osmotic conditions, euryhaline crustaceans incorporate ambient ions, mainly Na^+^ and Cl^−^, actively through chloride cells in the gills to maintain their hemolymph osmolality higher compared to the environment. Chloride cells in the posterior gills play a prominent role in the ionic regulation of hemolymph, and NKA, CAc, and NHE expressed in chloride cells are required to incorporate Na^+^ from environmental water to hemolymph. NKA is distributed in the basolateral membrane of chloride cells, which transports Na^+^ from the cytoplasm to the hemolymph and keeps the intracellular Na^+^ concentration relatively low, generating driving force of Na^+^ uptake. CAc in chloride cells catalyzes the formation of H^+^ and HCO_3_^‐^ HCO from H_2_O and CO_2_, and the derived H^+^ supports Na^+^ uptake through activation of NHE located in the apical membrane (Charmantier et al., 2009; Freire et al., 2008; Griffith, 2017; Henry et al., 2012). In addition, the expression of genes encoding NKA, CAc, and NHE in euryhaline crabs increases under hypo‐osmotic conditions (Henry et al., 2003; Liu et al., 2015; Lovett, et al., 2006; Lucu & Flik, 1999; Lucu & Towle, 2003; Pan et al., 2016). This study revealed that the expression of these genes was also enhanced in *H. tridens* and *M. japonicus* under hypo‐osmotic conditions (8.6 mmol/L NaCl solution) (Figure 5), consistent with previous studies. In addition, the expression of these genes in posterior gills also increased in both species in 513.3 mmol/L NaCl solution as in 8.6 mmol/L NaCl solution, strongly indicating that ambient minor cations decrease the expression of these genes under isosmotic conditions with seawater. The enhanced expression of these genes could account for the increased Na^+^ concentration and, concomitant with decreased K^+^ concentration, increased Na^+^/K^+^ ratio in hemolymph in 513.3 mmol/L NaCl solution. An imbalance between Na^+^ and K^+^ concentrations in hemolymph can cause an increase in the mortality rate in crustaceans (Sowers et al., 2006). Thus, the increased expression of the genes encoding NKA, CAc, and NHE in the most posterior gills might be the cause of death of *H. tridens* and *M. japonicus* reared in 513.3 mmol/L NaCl solution through an increased Na^+^/K^+^ ratio. The following findings support this view: (a) administration of acetazolamide and amiloride, inhibitors of CA and NHE, respectively, increased the survival rate of *H. tridens* and *M. japonicus* in 513.3 mmol/L NaCl solution (Figure 6), and (b) incubation of *C. dehaani* in 513.3 mmol/L NaCl solution, showing a high survival rate compared to *H. tridens* and *M. japonicus,* induced only limited increase of the expression of NKA α subunit and CAc and does not increase the Na^+^/K^+^ ratio (Figure 3).

It has been reported that the ambient minor cations in seawater are indispensable for the survival of euryhaline crustaceans. However, the physiological and ecological role of these ambient minor cations in euryhaline crustaceans is not completely understood. Mg^2+^ is involved in regulation of more than 300 enzymes, including NKA (Apell et al., 2017). In fact, activity of NKA in gills of crab is modulated by ambient Mg^2+^ (Antunes et al., 2017; Masui et al., 2005, 2009). Moreover, Ca^2+^ plays an important role in various biological processes, particularly in stabilizing biological membranes, increasing the tightness of intracellular tight junctions, and thereby controlling ion permeability across the gill epithelium in fish (McDonald & Milligan, 1988; McDonald et al., 1983). Lowering the concentration of ambient Ca^2+^ reduces hemolymph Na^+^ concentration, irrespective of environmental pH in crayfish *Cherax destructor* (Ellis & Morris, 1995). In addition, when ambient pH is low, decreased ambient Ca^2+^ concentration reduces plasma or hemolymph Na^+^ concentration in teleost species, *Salmo gairdneri* and *Oryzias latipes*, and crustaceans, *Daphnia magna* and *D. middendorffiana*, and leads to increased mortality (Havas et al., 1984; Jozuka & Adachi, 1979; McDonald et al., 1980). In this study, however, hemolymph Na^+^ concentrations in 513.3 mmol/L NaCl solution increased both in *H. tridens* and *M. japonicus* compared to that in 513.3 mmol/L NaCl + MCK solution (Figure 3). This suggests that the lethality in the 513.3 mmol/L NaCl solution was not because of an inhibition of NKA by removal of Mg^2+^. In addition, we showed that the role of Ca^2+^ for survival of *H. tridens* and *M. japonicus* was substitutable by Mg^2+^ (Figure 2). Furthermore, both these divalent cations could not overcome the lethality caused by removal of K^+^ (Figure 2), which is a striking contrast to *Oryzias latipes*, in which only the addition of Ca^2+^ increases their survival rate in low pH conditions (Jozuka & Adachi, 1979). These results strongly suggest that minor cations have different roles on these species from those described above, although it is possible that cell membrane integrity and cell junction formation in gills would also be affected by the 513.3 mmol/L NaCl solution also in these species.

This study suggests that ambient minor cations regulate the expression of specific genes in the gills, thereby affecting ion transport across the gills and hemolymph ionic composition. It might be necessary for euryhaline crustaceans inhabiting estuaries to sense subtle changes of ambient minor cation concentrations to adapt environmental salinity fluctuation. Future studies should be addressed to know whether these ambient minor cations directly affect gene expression in the gills. A comprehensive identification of the genes that are involved in ion transport and whose expression is regulated by ambient minor cations is also an important challenge.

### 4.3 Role of ambient minor cations under hypo‐osmotic conditions

This study revealed that ambient minor cations, especially K^+^, caused lethal damage to *M. japonicus* under hypo‐osmotic conditions (4.3 mmol/L NaCl solution) (Figure 7), showing a striking contrast to isosmotic conditions (Figure 2b). Ambient K^+^ was essential in isosmotic conditions, but harmful in hypo‐osmotic condition. Thus it appears that ambient K^+^ has an opposite influence on the survival of *M. japonicus* in an osmotic condition‐dependent manner. The hemolymph K^+^ concentration in *M. japonicus* increased when the crabs were bathed in 4.3 mmol/L NaCl + 1.2 MCK solution compared to 4.3 mmol/L NaCl solution (Figure 8f). Because the hemolymph Na^+^ concentration is the same in both solutions, the Na^+^/K^+^ ratio in hemolymph decreases in 4.3 mmol/L NaCl + 1.2 MCK solution. It is possible to assume that a decreased Na^+^/K^+^ ratio accounts for the lethality in *M. japonicus*. NKA and NKCC have been identified as molecules involved in K^+^ transport in chloride cells in the gills of euryhaline crustaceans (Charmantier et al., 2009; Freire et al., 2008; Griffith, 2017; Henry et al., 2012). NKA transports K^+^ from hemolymph to the cytoplasm in the opposite direction to Na^+^. On the other hand, NKCC is distributed in the apical membrane of chloride cells and incorporates K^+^ from the environment into cells, concomitant with Na^+^ and Cl^−^. Northern blotting showed that NKA α subunit expression was attenuated in all three species in the presence of ambient minor cations under hypo‐osmotic conditions (Figure 9). This decreased NKA α subunit expression might decrease K^+^ transport from hemolymph to chloride cells, possibly resulting in increased hemolymph K^+^ concentration. However, it is unclear whether this downregulation of NKA α subunit gene accounts for the increased hemolymph K^+^ concentration in *M. japonicus*, since this gene was also attenuated in *C. dehaani* in which ambient minor cations did not affect the hemolymph K^+^ concentration (Figure 8g). In addition, NKCC gene expression also decreases in the presence of ambient minor cations under hypo‐osmotic conditions in both *H. tridens* and *M. japonicus*, indicating that NKCC does not contribute to increased hemolymph K^+^ concentration, because decreased expression of this gene should lead to decreased hemolymph K^+^ concentration. It is possible that unidentified transporters and/or channels involved in K^+^ transport are activated by ambient minor cations to accelerate K^+^ uptake under hypo‐osmotic conditions.

Interestingly, in *H. tridens*, ambient minor cations increased the hemolymph K^+^ concentration in hypo‐osmotic condition (Figure 8e) although they caused no mortality (Figure 7a). Therefore, it is possible that *H. tridens* is more tolerant to elevated K^+^ concentrations and low Na^+^/K^+^ ratio in hemolymph compared to *M. japonicus*. It is noteworthy that the average hemolymph K^+^ concentration in *M. japonicus* bathed in 513.3 mmol/L NaCl + 1.2 MCK is 15.3 ± 1.28 mmol/L, which is significantly higher than the nominal K^+^ concentration in bathing media (13.4 mmol/L), although the average hemolymph K^+^ concentration in *H. tridens* is 11.5 ± 1.79 mmol/L, still less than the nominal K^+^ concentration in bathing media. Thus ambient minor cations may affect the K^+^ transport system differently in *H. tridens* and *M. japonicus*. Another possibility is that some unidentified damage occurs only in *M. japonicus* but not in *H. tridens* in the presence of ambient minor cations under hypo‐osmotic conditions. The fact that osmotic concentration increased significantly in the presence of minor cations in *M. japonicus* but not in *H. tridens* (Table 3) suggests that transport of some ions other than Na^+^ and K^+^ was affected only in *M. japonicus*. The mechanism by which ambient minor cations cause mortality to *M. japonicus* under hypo‐osmotic conditions should be investigated in the future.

### 4.4 Distinct response of *Chiromantes dehaani* to ambient minor cations

The response of *C. dehaani* to ambient minor cations is different from that of *H. tridens* and *M. japonicus*. First, under isosmotic conditions, the decrease of survival rate of *C. dehaani* in 513.3 mmol/L NaCl was less drastic (Figure 1). Consistent with this result, there was no significant difference in hemolymph Na^+^ and K^+^ concentrations between crabs bathed in 513.3 mmol/L NaCl and 513.3 mmol/L NaCl + MCK solutions (Figure 3c,f). In addition, NKA expression was enhanced but less drastic compared to *H. tridens* and *M. japonicus*, and CAc expression was not activated in 513.3 mmol/L NaCl solution (Figure 5). Furthermore, ambient minor cations did not affect the survival rate (Figure 7c) and hemolymph Na^+^ and K^+^ concentrations under hypo‐osmotic conditions (Figure 8c,g). These results strongly indicate that *C. dehaani* is less susceptive to ambient minor cations compared to *H. tridens* and *M. japonicus*. Compared to *H. tridens* and *M. japonicus*, *C. dehaani* is adaptive to fresh water and habitats of this species are not restricted within estuaries but extend more upstream regions (Irawan & Kijima, 1993; Kobayashi, 2000). Susceptibility to ambient minor cations would be less adaptive for such species and lost in *C. dehaani*. Alternatively, the difference of sensitivity to ambient minor cations is due to phylogenetic difference of these species. Comparative studies using other fresh water‐adaptive crabs, such as *Eriocheir japonica* and *E. sinensis* (Varunidae), will elucidate the relationship between sensitivity to ambient minor cations and adaptation to fresh water.

## 5 CONCLUSION

Ambient minor cations regulate the expression patterns of specific genes involved in ion transport and thereby affect the hemolymph ionic composition and, ultimately, the survival of *H. tridens* and *M. japonicus*. In contrast, in *C. dehaani*, which lives in more upstream region, ambient minor cations hardly affect the hemolymph ionic composition, likely because of less susceptibility of gene expression to ambient minor cations. The different response to ambient minor cations between euryhaline species could be due to the difference in the microenvironment of their habitats or phylogenetic difference.

## CONFLICT OF INTERESTS

The authors declare that there are no conflicts of interests.

## AUTHOR CONTRIBUTIONS

**Masahiro Yamaguchi:** Conceptualization (lead); formal analysis (lead); funding acquisition (lead); investigation (lead); writing–original draft (lead); writing–review and editing (lead). **Kouichi Soga:** Investigation (supporting); writing–review and editing (supporting).

### Open Research Badges

This article has earned an Open Data Badge for making publicly available the digitally‐shareable data necessary to reproduce the reported results. The data is available at https://doi.org/10.6084/m9.figshare.12788132.

## Data Availability

The sequences of isolated cDNAs in this study were registered in GenBank (accession numbers: LC214855, NKA α subunit of *H. tridens*; LC375964, CAc of *H. tridens*; LC572286, NHE of *H. tridens*; LC572287, NKCC of *H. tridens*; LC214856, NKA α subunit of *M. japonicus*; LC375965, CAc of *M. japonicus*; LC572288, NHE of *M. japonicus*; LC572289, NKCC of *M. japonicus*; LC572290, NKA α subunit of *C. dehaani*; LC572291, CAc of *C. dehaani*; LC572292, NHE of *C. dehaani*; LC572293, NKCC of *C. dehaani*). Raw data are publicly available on figshare (https://doi.org/10.6084/m9.figshare.12788132).
